# Effect of Personal Characteristics on Individual Support for Indoor Smoke-Free Air Laws, Indiana, 2008

**DOI:** 10.5888/pcd9.120091

**Published:** 2012-10-04

**Authors:** Terrell W. Zollinger, Robert M. Saywell, Joshua J. Robinson, Stephen J. Jay, Miranda H. Spitznagle

**Affiliations:** Author Affiliations: Terrell W. Zollinger, Stephen J. Jay, Indiana University School of Medicine, Indianapolis, Indiana; Joshua J. Robinson, US Department of Veterans Affairs, Washington, District of Columbia; Miranda H. Spitznagle, Indiana State Department of Health, Indianapolis, Indiana.

## Abstract

**Introduction:**

Policy makers should understand the attitudes and beliefs of their constituents regarding smoke-free air legislation. The purpose of this study was to evaluate the effect of selected personal characteristics on attitudes and beliefs about secondhand smoke in Indiana and on support for smoke-free air laws.

**Methods:**

Data were obtained from the 2008 Indiana Adult Tobacco Survey of 2,140 adults and included 11 sociodemographic variables. Chi-square and multiple logistic regression analyses were used to test for significant associations between sociodemographic characteristics and support for statewide or community smoke-free air legislation.

**Results:**

Most respondents (72.3%) indicated that they supported laws making work places smoke-free. After adjusting for the effects of the other variables, 3 were found to be significant predictors of support: being a never or former smoker, being female, and being aware of the health hazards of secondhand smoke. Age, race/ethnicity, income, urban or rural county of residence, employment status, and having children in the household were not significant when adjusting for the other characteristics.

**Conclusion:**

Most Indiana residents support smoke-free air legislation for workplaces. The support was constant among most groups across the state, suggesting policy makers would have the backing of their constituents to pass such legislation. The results of this study suggest that efforts to gain support for smoke-free air laws should focus on men, people unaware of the health hazards from secondhand smoke, and smokers and former smokers.

## Introduction

The health effects of cigarette smoking became widely recognized after publication of the 1964 US Surgeon General’s report that identified smoking as a major health hazard (1). Smoking remains the leading cause of preventable death and disease in the United States, being responsible for approximately 1 in 5 deaths annually or about 443,000 premature deaths per year during 2000–2004. Cigarette smoking costs more than $193 billion annually ($97 billion in lost productivity plus $96 billion in annual economic health expenditures) (2). Despite the evidence of negative health effects, 17.3% of the US population and 21.2% of Indiana residents were smokers in 2010 (3). In Indiana during 2010, an estimated 9,700 premature deaths were attributable to smoking, not counting secondhand smoke (SHS) exposure or burns. In addition, smoking-attributable health care expenditures totaled $4.7 billion in annual health and other economic costs, including $487 million in Medicaid payments (4).

SHS poses a high health risk to nonsmokers through exposure at home, in the workplace, and in public areas such as bars, restaurants, and recreation venues. SHS is dangerous to the health of the public and especially to children (5). Numerous studies have linked adult exposure to SHS with an increased risk of lung cancer, nasal sinus cancer, breast cancer, cervical cancer, ischemic heart disease, stroke, eye and nasal irritation, spontaneous abortions, and asthma leading to premature death in adult nonsmokers (1,5). The health effects of SHS in children include asthma and an increased risk for low birthweight, sudden infant death syndrome, preterm delivery, several childhood cancers, respiratory problems, and ear infections (5). Other studies have identified the relationship between childhood health and exposure to SHS, which differs by parental smoking status, prenatal and postnatal smoking patterns, and the location of SHS exposure (6–8). Exposure to SHS is a serious problem, but the long-term trend of decreasing exposure to SHS among children has declined during the past decade. Complete home smoking bans in US households with children and smokers increased from 14.1% in 1992–1993 to 50% in 2006–2007 (9). However, sex and racial/ethnic disparities continue to exist, and household bans were less common among smoking families with older children and in African American households (9).

SHS costs approximately $10 billion annually in the United States for health care expenditures, illness, and death (10). Zollinger et al found that the overall cost of health care and premature death attributed to SHS exposure for Indiana residents was estimated to be $1.3 billion in 2010, resulting in SHS-related costs of $201 per capita. Of the $1.3 billion, $977 million was from premature death ($879 million for adults and $98 million for children) (11).

Communicating the effect of SHS on the costs of health care and premature death to the public may increase support for smoke-free air laws. The public’s recognition of the health hazards from exposure to SHS and the estimated economic impact of SHS have changed the public’s attitude toward SHS, leading to increased efforts to encourage enactment of smoke-free air legislation (4). The number of smoke-free air laws in Indiana communities increased from 2001 to 2011 from having no local community smoke-free air laws in 2001 to 33 city and county smoke-free air laws in place in 2011, protecting 37.8% of the state’s approximately 6 million people from SHS (4,12). Despite these favorable trends, Indiana was 49th on the list of 50 states for protection of workers from smoking at worksites (13).

Several studies have demonstrated that various factors may influence the level of public support for worksite smoke-free air laws. These factors include an awareness of the benefits from these laws such as a better understanding of the health effects of SHS, the perception that smoke-free air laws and not smoking have become normative behavior, the recognition that smoke-free air laws reduce exposure to SHS, and awareness that smoke-free air laws may be beneficial to community businesses (14–19).

Despite increasing public awareness of the benefits of smoke-free air laws, opposition to such reforms persists among the public and policy makers. Opposition to or support for smoke-free air laws may be influenced by race/ethnicity, education, presence of other smokers, and the presence of children in the home (20–28). The purpose of this study was to evaluate the effect of selected personal characteristics on attitudes and beliefs about SHS in Indiana and on support for smoke-free air laws. This information will help lawmakers create more effective legislation to reduce SHS exposure.

## Methods

We used data from the Indiana Adult Tobacco Survey (ATS) conducted during 2008 (29). This telephone-based survey was designed as a random sample of adults in Indiana aged 18 or older. African American and Hispanic adults were oversampled as were adults in rural regions of the state. The 2,140 survey participants who answered the 2 questions regarding their support for clean air workplace laws were included in the study. The survey gathered information on demographic variables and attitudes about SHS exposure policies, perceptions, and beliefs.

The demographic data included age, race, ethnicity, sex, household income, county of residence, education level, employment status, and the number of children in the household. Other related tobacco use and exposure information included the number of adult smokers in the household and smoking history to determine whether the respondent was a current smoker, former smoker, or never smoker.

Responses to the race and ethnicity questions were combined to create 4 categories: white non-Hispanic, black non-Hispanic, other non-Hispanic, and Hispanic/Latino. Age and household income questions included categories for response options. The county of residence was used to determine the urban/rural resident status of each respondent by using a rural-urban continuum code scheme developed by the US Department of Agriculture (30). Education level was classified as less than high school graduate, high school graduate, some college or associate’s degree, college graduate, and some postgraduate school. Employment status data were coded as currently employed or currently unemployed.

Two survey questions asked about the participants’ support for no-smoking laws in indoor workplaces at the state or local community level. The responses were merged to classify a person as one who would support or not support smoke-free air policies for all indoor workplaces, including casinos, restaurants, and bars.

Institutional review board approval was obtained from Indiana University. We used SPSS version 18.0 (IBM, Chicago, Illinois) and SAS version 9.1 (SAS Institute, Inc, Cary, North Carolina) for statistical analyses.

The data were weighted to account for the sampling scheme before data analysis. We used the χ^2^ test to perform bivariate associations between the demographic and smoking status data and the dependent “support” variable. Multiple logistic regression analyses were also performed to assess the effect of each variable while controlling for the effect of other variables. Significance was set at *P <* .05 for the bivariate analysis and logistic regression.

## Results

In 2008, approximately 81% of all households were smoke-free, and 55% of the households where any resident was a smoker did not allow smoking inside the house. Approximately 73% of indoor workers reported that their workplace was 100% smoke-free, and there was widespread support for smoke-free workplaces even among current smokers. Approximately 82% of smokers and 95% of nonsmokers thought that smoking should not be allowed in indoor work areas. In addition, 72.3% of the respondents supported community or statewide smoke-free air legislation that would eliminate tobacco smoke from all indoor work areas (29).

After weighting the data, approximately one-half of the data used in the analysis were from women and four-fifths were from white non-Hispanics. Two-thirds were aged 30 to 64 (31.2% were 30 to 44 and 33.0% were 45 to 64). One-third reported an income of $75,000 per year or more, and more than one-fourth had an income of less than $34,000. Almost one-third had a college degree or higher education. Additionally, 65.4% of the respondents were currently employed. Three-quarters were living in an urban county. More than one-half reported not having any children in the household. One-quarter reported that no adult smokers were present in their household. Almost all of the respondents considered SHS to be a health hazard. Two-fifths of the respondents reported being former (25.3%) or current (16.6%) smokers.

Bivariate analyses revealed that 6 of the 11 study variables were significant, indicating that the respondents more likely to support smoke-free air laws were those with more education, women, the youngest (aged 18–29) and oldest (aged ≥65), former and never smokers, those without adult smokers in the household, and those agreeing that SHS is hazardous to one’s health (Table 1). 

**Table 1:** Respondents Who Support Indoor Smoke-Free Air Laws by Personal Characteristics (n = 2,140), 2008 Indiana Adult Tobacco Survey

Characteristic	n (%)	χ^2^ *P* value
**Race/ethnicity**		
White non-Hispanic	1,316 (72.5)	.51
Black non-Hispanic	119 (71.3)	
Other non-Hispanic	17 (70.8)	
Hispanic/Latino	70 (79.5)	
**Annual income, $**		
<25,000	237 (66.4)	.18
25,000–34,999	147 (71.0)	
35,000–49,999	265 (71.8)	
50,000–74,999	298 (73.6)	
≥75,000	468 (73.1)	
**Education**		
<High school graduate	76 (60.3)	<.001
High school graduate	495 (69.6)	
Some college or associate’s degree	473 (71.9)	
College graduate	315 (75.5)	
Some postgraduate school	179 (85.6)	
**Urbanicity**		
Urban	1,187 (72.9)	.25
Rural	360 (70.3)	
**Sex**		
Female	839 (76.4)	<.001
Male	708 (67.9)	
**Age, y**		
18–29	294 (75.8)	.004
30–44	485 (72.7)	
45–64	490 (67.9)	
≥65	273 (76.9)	
**Smoking status**		
Never smoker	1,060 (85.2)	<.001
Former smoker	374 (69.1)	
Current smoker	113 (31.8)	
**Employed**		
Yes	997 (72.4)	.94
No	530 (72.5)	
**Children present in household**		
Yes	664 (71.9)	.71
No	882 (72.6)	
**Adult smokers present in household**		
Yes	278 (58.8)	<.001
No	1,061 (77.1)	
**Secondhand smoke is health hazard**		
Yes	1,542 (74.4)	<.001
No	4 (6.3)	

Logistic regression analyses revealed significant odds ratios (ORs) in 3 of the 11 study variables (Table 2): women were more than 1.5 times as likely as men, never smokers were almost 9 times as likely as current smokers, and former smokers were more than 4.5 times as likely as current smokers to support indoor smoke-free air legislation. People who were aware that SHS is hazardous to one’s health were 34 times as likely as those who were unaware of the hazards to support indoor smoke-free air legislation.

**Table 2:** Logistic Regression Analysis of the Support for Indoor Smoke-Free Air Laws by Personal Characteristics (n = 2,140), 2008 Indiana Adult Tobacco Survey

Characteristic	OR (95% Confidence Interval)
**Race/ethnicity**	
White non-Hispanic	1 [Reference]
Black non-Hispanic	0.65 (0.34–1.26)
Hispanic/Latino	1.68 (0.66–4.29)
**Annual income, $**	
<25,000	1 [Reference]
25,000–34,999	1.19 (0.55–2.60)
35,000–49,999	1.05 (0.53–2.10)
50,000–74,999	1.06 (0.54–2.05)
≥75,000	0.98 (0.52–1.84)
**Education**	
<High school graduate	1 [Reference]
High school graduate	0.72 (0.30–1.69)
Some college or associate’s degree	0.88 (0.36–2.14)
College graduate	0.81 (0.31–2.14)
Some post-graduate school	1.09 (0.40–3.01)
**Urbanicity**	
Urban	1.21 (0.75–1.93)
Rural	1 [Reference]
**Sex**	
Female	1.70 (1.15–2.50)
Male	1 [Reference]
**Age, y**	
18–29	1 [Reference]
30–44	0.94 (0.48–1.86)
45–64	0.57 (0.30–1.06)
≥65	0.75 (0.34–1.65)
**Smoking status**	
Never smoker	8.99 (5.26–15.37)
Former smoker	4.69 (2.70–8.14)
Current smoker	1 [Reference]
**Employed**	
Yes	0.91 (0.56–1.46)
No	1 [Reference]
**Children present in household**	
Yes	0.76 (0.48–1.21)
No	1 [Reference]
**Adult smokers present in household**	
Yes	1 [Reference]
No	1.28 (0.81–2.03)
**Secondhand smoke is health hazard**	
Yes	34.23 (11.32–103.54)
No	1 [Reference]

## Discussion

Our findings indicate that, after adjusting for other personal characteristics, former and never smokers, women, and those who believe that smoking is hazardous to one’s health were more likely to support smoke-free air laws. We found that support for smoke-free air policies did not differ by age, race/ethnicity, education level, urban or rural location, income level, employment status, or composition of the household. Smoke-free air laws are spreading throughout the world (31), and although this process has been under way in the United States for more than 30 years, it is not yet complete. In spite of the advantages, many states and communities have not implemented SHS laws (32).

Reluctance to pass smoke-free air legislation may be influenced by several factors, including questions concerning the scientific merit of studies promoting the benefits of eliminating public smoking, reluctance to challenge the “right to smoke” advocates, and lack of awareness of support for smoke-free air legislation in the general population. In Colorado, support for clean indoor air policies was prevalent among public officials who believed that tobacco use is a problem in their community, believed that SHS is a problem, and believed that city and county government should be involved in people’s decisions about smoking (33).

Knowing the demographic characteristics of the people who are more likely not to support the passage of smoke-free air ordinances and helping them become better informed of the health and economic benefits arising from smoke-free air legislation may increase the likelihood of their passage. Several studies have reported on attitudes toward SHS laws by smoking status and demographic characteristics (20–28). Predictors of support for SHS laws are age of adult/child in the household (24,25,27), race of adult/child in the household (24,26,28), ethnicity of adult/child in the household (24,28), smoking status (25–27), education (24,26,27), income level (26), sex (25–28), presence of household smokers (21), voluntary in-home smoking ban (27), exposure to SHS at work (26), having nonsmoking friends (27), living in an area with smoke-free air laws (27), and sexual orientation (28).

Knowledge that SHS is a health hazard to nonsmokers is a strong predictor of support for smoke-free air legislation. In Indiana, the 2008 ATS survey revealed that most respondents believed that SHS was a health hazard, and activities to inform the public of the risks from SHS exposure seem to be effective in gaining public support for smoke-free air legislation (34).

The benefits from smoke-free air policies are not limited to improved health of nonsmokers. Smoke-free air laws also encourage those who would like to quit smoking. These laws can be used to further educate the public about the benefits of a smoke-free air act and may increase the support from current smokers who are contemplating smoking cessation (29).

There are several strengths to this study, including a large sample size, random selection of study participants, and the use of a standard survey instrument and protocol to collect the data. However, there are several limitations. First, the data used for analyses were collected through a telephone survey. Thus, respondents without a home telephone were excluded. The data were self-reported and subject to the inaccuracies inherent in asking sensitive behavior questions, such as underestimating one’s smoking status. As such, misclassification may have occurred in several variables and is dependent on the person responding to the survey. We believe the effects of these limitations would not affect the outcome; however, findings should be interpreted with caution.

In Indiana, the community leaders, media, and policy makers need to understand the level of public support for smoke-free air policies that was evident from this study. Policy makers, in turn, need to act on behalf of their constituents to support smoke-free air laws because these policies are supported by an extensive body of research that has documented their value in improving public health. The findings in this study could guide the development and implementation of comprehensive smoke-free air policies in the workplace in Indiana and elsewhere.

In addition to promoting the benefit of SHS laws, this study further demonstrates the various demographic characteristics of the supporters of SHS laws in other states and communities. Such variation suggests that states may benefit from identifying the individual characteristics of their citizens who may not be supportive of SHS laws; the data may enable states to tailor interventions to increase support for SHS laws.

Legislators and policy makers need to be convinced that the level of public support for clean indoor air policies is high and consistent across a broad cross-section of their constituents. Using such findings could be instrumental in developing and implementing smoke-free air legislation in the workplace. Such legislation, taking into account the economic and health effects of smoking and SHS, can potentially save millions of dollars in health expenses and prevent premature death. Further research is needed to better understand the determinants of policy makers’ support of and action on smoke-free air legislation.
